# Awareness and consciousness in humans and animals – neural and behavioral correlates in an evolutionary perspective

**DOI:** 10.3389/fnsys.2022.941534

**Published:** 2022-07-14

**Authors:** Günter Ehret, Raymond Romand

**Affiliations:** ^1^Institute of Neurobiology, University of Ulm, Ulm, Germany; ^2^Faculty of Medicine, Institute de Génétique et de Biologie Moléculaire et Cellulaire (IGBMC), University of Strasbourg and Centre National de la Recherche Scientifique (CNRS), Strasbourg, France

**Keywords:** brain energy costs, brain waves, EEG, ERP, event-related potentials, gamma-band activity, neural correlates of consciousness, selective attention

## Abstract

Awareness or consciousness in the context of stimulus perception can directly be assessed in well controlled test situations with humans via the persons’ reports about their subjective experiences with the stimuli. Since we have no direct access to subjective experiences in animals, their possible awareness or consciousness in stimulus perception tasks has often been inferred from behavior and cognitive abilities previously observed in aware and conscious humans. Here, we analyze published human data primarily on event-related potentials and brain-wave generation during perception and responding to sensory stimuli and extract neural markers (mainly latencies of evoked-potential peaks and of gamma-wave occurrence) indicating that a person became aware or conscious of the perceived stimulus. These neural correlates of consciousness were then applied to sets of corresponding data from various animals including several species of mammals, and one species each of birds, fish, cephalopods, and insects. We found that the neural markers from studies in humans could also successfully be applied to the mammal and bird data suggesting that species in these animal groups can become subjectively aware of and conscious about perceived stimuli. Fish, cephalopod and insect data remained inconclusive. In an evolutionary perspective we have to consider that both awareness of and consciousness about perceived stimuli appear as evolved, attention-dependent options added to the ongoing neural activities of stimulus processing and action generation. Since gamma-wave generation for functional coupling of brain areas in aware/conscious states is energetically highly cost-intensive, it remains to be shown which animal species under which conditions of lifestyle and ecological niche may achieve significant advantages in reproductive fitness by drawing upon these options. Hence, we started our discussion about awareness and consciousness in animals with the question in how far these expressions of brain activity are necessary attributes for perceiving stimuli and responding in an adaptive way.

## Introduction

How to scientifically approach animal consciousness when human consciousness has already been named *an ill-defined explanandum* (Singer, 2019), i.e., an ill-defined term to be explained scientifically? Hence, it is not surprising that 29 theories (and various definitions) of consciousness in humans have been identified in an analysis of 68 studies that remained suitable from a scan of 1130 articles published between 2007 and 2017 (Sattin et al., 2021). In many of the publications, consciousness was defined *as being aware of something or as the subject experience* (Sattin et al., 2021).

In the subjective experience of humans, consciousness can be found attached to basically three domains of neural processing, (a) the detection and perception of the content of sensory input from outside and inside the own body, (b) the cognition-based preparation for actions, and (c) the observation and/or intentional control of own ongoing thoughts and emotional/motivational states (e.g., Dehaene and Naccache, 2001; Zeman, 2001; Raffone and Pantani, 2010; Dehaene and Changeux, 2011; Brown et al., 2019). Points (a) and (b) relate to phenomenal and access consciousness, respectively (Block, 2005), point (c) relates to bodily self-awareness, introspective awareness, and metacognition (e.g., Metcalfe, 2008; DeGrazia, 2009; Terrace and Son, 2009). Since we know much about the neurobiology of stimulus processing and perception in animals, we will concentrate in our present account on aspect (a), and will name the subjectively experienced stimulus perception as *becoming aware of something*, i.e., “awareness.” We will use the term “consciousness” in contexts of aspects (b) and (c) and consider these aspects when they help to interpret animal data obtained from the evaluation of (a). Our analysis will show that the thus defined differentiation between stimulus-related awareness and cognitive action-related consciousness has neural correlates in measures of brain activity.

In our present use of the term “awareness” we make a distinction to the use of the terms “sentience,” “primitive awareness” and “minimal consciousness” as they sometimes occurred in the context of discussions about consciousness (e.g., Merker, 2007; Denton et al., 2009; Bronfman et al., 2016). We relate these terms to instinctive behavior, or goal-directed behavior in the context of instincts, i.e., inherited, rather stereotyped and stimulus-guided selection of actions, which can function without implying both subjective awareness about the content of perception and consciousness about the action as necessary variables (see later paragraphs on awareness and consciousness in animals).

So far, possible awareness and/or consciousness in a given animal has been inferred from its brain anatomy and physiology in relation to structures and processes in human brains known to support consciousness, and from behavior and cognitive abilities which, in humans, are closely related to awareness and/or consciousness (e.g., Griffin, 2000; Seth et al., 2005; Edelman and Seth, 2009; Damasio, 2010; Boly et al., 2013; Mashour and Alkire, 2013; Fabbro et al., 2015; Bronfman et al., 2016; Le Neindre et al., 2017; Pennartz et al., 2019; Birch et al., 2020a,b; Irwin, 2020; Nieder et al., 2020; Ben-Haim et al., 2021; Kaufmann, 2021; Mallatt and Feinberg, 2021). In our present neuroscience approach, we follow the search for neural correlates of consciousness (NCCs) in brain activity of humans, a main topic of human neuroscience for many years (e.g., Crick and Koch, 1990; Dehaene and Naccache, 2001; Gaillard et al., 2009; Aru et al., 2012b; Koch et al., 2016; Tononi et al., 2016; Seth, 2018; Owen and Guta, 2019; Rowe et al., 2020; Dembski et al., 2021; Lepauvre and Melloni, 2021; Sergent et al., 2021). The understanding of how awareness and consciousness may emerge from brain activity is not only essential to bridge the gap from subjective experience to brain mechanisms in humans. If neural markers in activity patterns of the human brain reliably reported about awareness and/or consciousness, this knowledge could be used as a new objectifiable gate for getting experimentally reproducible access to study the possible presence of awareness and consciousness in animals.

Therefore, we analyze and discuss response data from the human brain concerned with neural signatures of aware and/or conscious processing in the light of recent progress and search for such neural markers in comparable studies on animals. We also discuss with several examples from different stages in evolution, in which conditions animals may perceive and act non-consciously, and in how far at all animals *need* to be subjectively aware and, possibly, conscious of the stimuli they perceive and respond to. Brain waves in humans and animals show that, after stimulus onset, awareness, as a neural processing *option*, may come first and then consciousness as another *option* may follow. The same order may become apparent in the evolutionary perspective.

## In search for neural markers of awareness and consciousness in human brain activity

In humans, basis conditions for generating awareness and consciousness comprise sufficient activation in the ascending reticular activation system (ARAS) to induce and support arousal and wakefulness (e.g., Parvizi and Damasio, 2001; Edlow et al., 2012; Lewis et al., 2015), and sufficient activation in cortico-thalamic loops in order to stabilize the cortical stimulus representation (e.g., Newman and Baars, 1993; Seth and Baars, 2005; Edelman and Seth, 2009; Dehaene and Changeux, 2011; Ward, 2011; Nani et al., 2019). Therefore, we assume that awareness and consciousness in animals also critically depend on sufficient arousal and wakefulness and on activation of neural loops in higher brain centers in order to stabilize the stimulus representation for awareness/consciousness to be generated.

NCCs in sensory processing and perception have extensively been studied using event-related potentials (ERPs) and brain wave synchrony [electroencephalographic (EEG) measurements] with adult humans who were able to report (verbally or via instrumental behavior) about their awareness of the experimental stimuli in various experimental settings (e.g., Sergent et al., 2005; Del Cul et al., 2007; Bekinschtein et al., 2009; Lamy et al., 2009; Dehaene and Changeux, 2011; Silverstein et al., 2015; Sanchez et al., 2020; Eklund et al., 2021). In such recordings, NCCs of three neural processes may be identified (Aru et al., 2012b; de Graaf et al., 2012; Li et al., 2014) – those preceding or leading to the conscious experience, those representing the conscious experience, those following the conscious experience (e.g., the report about the experience). In order to separate NCCs of awareness (domain (a), see Introduction) from those of following conscious actions (domain (b), see Introduction), several experiments without or with modified/controlled task requirements have been reported in studies on humans (e.g., Pitts et al., 2014b; Tsuchiya et al., 2015; Koivisto et al., 2016; Ye et al., 2019; Cohen et al., 2020; Mazzi et al., 2020; Rowe et al., 2020; Faramarzi et al., 2021; Schröder et al., 2021) and also in a recent study on macaque monkeys (Kapoor et al., 2022).

### With regard to event-related potentials as possible neural correlates of consciousness

**FIGURE 1:**
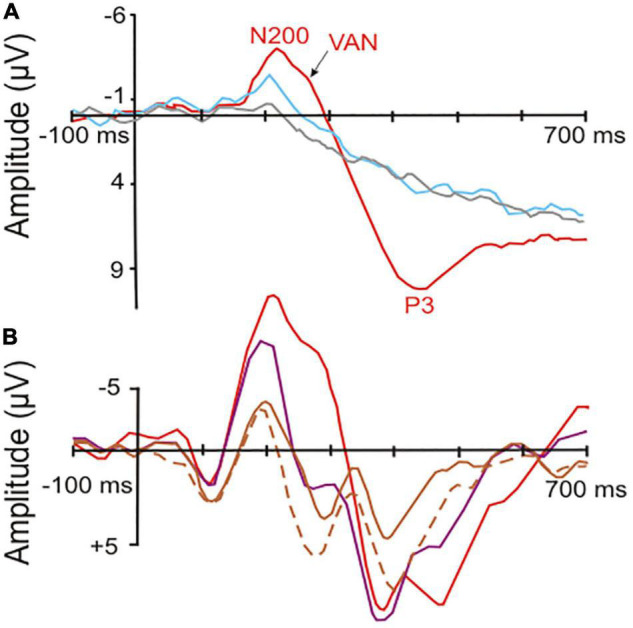
Event-related potentials (ERPs) in response to visual stimuli (starting at the point of origin) in human observers. **(A)** Potentials recorded from surface electrodes above the occipital cortex contralateral to the stimulated visual field. Red curve, correct response with high-awareness; blue curve, correct response reported without awareness; gray curve, incorrect response without awareness. The red and blue curves show significant visual awareness with peaks near 200 ms latency (N200, visual awareness negativity, VAN) after stimulus onset. In addition, the red curve shows a so called P3 (positive peak later than 300 ms after stimulus onset), which is related to a conscious response [modified from Koivisto and Grassini (2016); part of their Figure 2]. **(B)** Potentials recorded from surface electrodes above the left-side posterior-temporal cortex to stimuli in the center of the visual field. Red curve, high-awareness with behavioral response to relevant stimulus; purple curve, low-awareness without response to irrelevant stimulus; brown curves, looking at masked relevant (solid line) or masked irrelevant stimulus (dashed line) without response requirement. The visual awareness negativities (peaks near 200 ms latency after stimulus onset) increase with increasing visibility and relevance of the stimuli corresponding to a respective increase in the awareness of the stimuli. Only the red curve shows a broad P3-like peak in the latency range of about 350–500 ms, possibly related to the required (conscious) behavioral response [modified from Koivisto and Revonsuo (2008); their Figure 7 at T5 of the left side].

The following general picture emerged (e.g., Gaillard et al., 2009; Railo et al., 2011; Castelhano et al., 2013; Pitts et al., 2014b; Koivisto et al., 2016; Ye et al., 2019; Dembski et al., 2021; Faramarzi et al., 2021; Jimenez et al., 2021): Brain waves following the onset of stimuli (visual, auditory, or somatosensory) with less than about 180 ms latency relate to unconscious processing in sensory cortical areas. These waves include the mismatch-negativity with a peak between around 150–200 ms after stimulus onset, a wave signaling a deviation within a series of stimuli (e.g., Näätänen et al., 2004; Harms et al., 2016). A negative-going wave (waveform depending on electrode locations and stimulus modality) peaking around 200–250 ms after stimulus onset correlates with visual (V), auditory (A), or somatosensory (S) awareness of the stimuli, even if illusionary (Faramarzi et al., 2021). These awareness negativities (AN; i.e., VAN, AAN, SAN; see examples in Figure 1) are lateralized in the brain, i.e., their amplitudes are highest contralateral to the side of the sensory input (Koivisto and Grassini, 2016; Dembski et al., 2021; Eklund et al., 2021). Thus, they reflect neural processing rather closely related to the modality-specific sensory cortical representations.

Importantly, the occurrence of the ANs seems to indicate only a necessary, but not a sufficient, brain activation for generating awareness. In several visual perception paradigms, a VAN was present when the person was subjectively unaware of the stimulus (Railo et al., 2011; Tsuchiya et al., 2015; Koivisto and Grassini, 2016; Mazzi et al., 2020; compare Figure 1). In other words, VANs signal the potential to become aware which, however, does not automatically lead to awareness of the perceiving person. The probability of awareness of the person and with it the VAN amplitude increased with the degree of selective (focused) attention the person directed to the stimulus (Koivisto and Revonsuo, 2008, 2010; Koivisto and Grassini, 2016; compare Figure 1). Similarly, processing of visual stimuli (letter sequences) in studies of implicit learning elicited an ERP peak near 250 ms with implicit (unconscious) learning compared to non-learning (Fu et al., 2013). This shows again that the brain activity in a time window of about 200–250 ms after stimulus onset can be used as an indicator of awareness that may or may not become effective. Whether the person actually experienced awareness seems to depend on the directed attention. In other words, awareness of a stimulus appears as the perceptual target to be reached through selective attention. Attention has been found to be necessary for awareness of details of something (van Boxtel et al., 2010) or of something at all to be reached (Dehaene and Naccache, 2001; Cohen et al., 2012). Therefore, the level of selective attention, initiated either by the stimulus itself (bottom-up effect) or by expectation of the stimulus in a given context (top-down effect) is an important modulator of awareness to become effective (Nani et al., 2019; Sikkens et al., 2019).

More global brain activation starts about 250 ms after stimulus onset and may continue with various waveforms (e.g., late positive potentials) for more than 700 ms (compare Figure 1). These late positive potentials are often summarized under the term P3 or P300 (e.g., Pitts et al., 2014a,b; Koivisto et al., 2016; Cohen et al., 2020; Mazzi et al., 2020; Schröder et al., 2021), reflecting the influence of focused attention, working memory, confidence about perception, decisions and preparations for tasks or, in general, the further neural handling of the stimulus input on a noetic (Fabbro et al., 2015) or metacognitive (Fernandez-Duque et al., 2000; Shimamura, 2000) level. This handling can be conscious or non-conscious. Importantly, about 250–300 ms after stimulus onset, the dynamics of brain activation suggest a bifurcation in further conscious (global) or non-conscious processing (Gaillard et al., 2009; Sergent et al., 2021).

### With regard to EEG waves as possible neural correlates of consciousness

**FIGURE 2:**
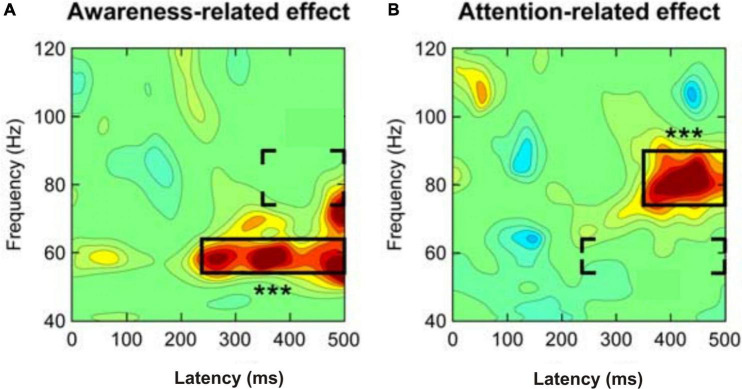
Separation of **(A)** awareness-related and **(B)** attention-related gamma-band activity when humans respond to a faint visual stimulus. Whether attended or not, a stimulus perceived with awareness induced mid-frequency gamma-band activity (about 54–64 Hz) at a latency of about 240 ms after stimulus onset (left panel). If spatial (selective) attention was directed to the stimulus, high-frequency gamma-band activity (about 76–90 Hz) occurred at a latency of about 350 ms after stimulus onset (right panel). In both panels the three stars indicate the highly significant effects in the framed areas. The brackets indicate the areas of a possible attention-related effect added to the awareness effect (left panel) or an awareness-related effect added to the attention effect (right panel). [Modified from Wyart and Tallon-Baudry (2008); their Figure 3C].

Cognitive acts including the conscious perception of stimuli and the conscious planning of actions are based on the integration of information via high synchrony of neuronal activation over large scales of the human cortex (Varela et al., 2001). The synchrony of neuronal activation can be measured as brain-wave prevalence and synchrony between waves in various frequency bands in the EEG (Singer, 1999). This also applies to animals (Engel and Singer, 2001). The comparability of brain waves has been shown for mammals and suggested also for birds (Buzsáki et al., 2013). The following essence can be distilled from the data and summaries in numerous studies on humans (e.g., Edwards et al., 2005; Canolty et al., 2006; Melloni et al., 2007; Gaillard et al., 2009; Dehaene and Changeux, 2011; Arnal and Giraud, 2012; Aru et al., 2012a; Castelhano et al., 2013; Li et al., 2014; Pitts et al., 2014a; Fries, 2015; Faramarzi et al., 2021): Local gamma-band activity (EEG wave frequencies of >30 Hz), for example from the occipital cortex for visual input stimuli or from the temporal cortex for auditory stimuli, may have been induced in the processing of the respective input stimuli from sensory detection to awareness. At about 200–250 ms after stimulus onset, strong gamma-band activity coincides with awareness becoming effective, especially when attention was directed to the stimuli (Bosman et al., 2012). Importantly, the stimulus-induced gamma-band activity being related to the possible awareness of the stimuli started earlier than further gamma-band activity in a higher frequency band related to selective attention directed to the stimuli about 350 ms after stimulus onset (Wyart and Tallon-Baudry, 2008; Figures 2A,B). Therefore, *local* gamma-band activity in sensory cortical areas starting about 200–250 ms after stimulus onset appears to correlate with possible awareness of stimuli, but seems not to be a sufficient marker of conscious perception.

Additional gamma-band activity occurring later than about 250 ms after stimulus onset and related to the onset of selective attention – in the Wyart and Tallon-Baudry (2008) study selective spatial attention started about 350 ms after stimulus onset (Figure 2B) – may support the stimulus processing toward consciousness. This later stimulus-induced and attention-supported gamma-band activity often concerned larger brain areas including, besides sensory and multimodal-integrative areas, parts of the frontal cortex (Melloni et al., 2007; Gaillard et al., 2009; Fries, 2015; Nani et al., 2019). Therefore, this gamma-band activity being related to more *global* processing seems to be characteristic for conscious perception with the option to respond accordingly.

### In conclusion

Collectively, these studies in humans have shown that it is possible to separately identify NCCs related to awareness and others related to consciousness in the course of stimulus perception. Early stimulus processing in the central nervous system happens while the persons are unaware of the stimuli. Then awareness of the stimuli may be initiated as a rather locally (in rather modality-specific neural substrates) generated and attention-supported phenomenon of brain activity. Then consciousness of the stimulus may follow, supported by selective attention and expressed by a more globally generated neural activity and by EEG gamma-waves which indicate synchronization in large neural networks. Conscious stimulus perception needs at least 250–300 ms time after stimulus onset to be realized on a cognitive level. The immediately preceding stimulus-related brain activity is associated with the state of awareness. Awareness seems to be a necessary, but not a sufficient, prerequisite for conscious stimulus perception. Importantly, both awareness of and consciousness about perceived stimuli appear as phenomena of brain activation added after the initial stimulus analysis, as attention-dependent *options*, to the ongoing neural activities of further stimulus processing and action generation.

The described distinction between awareness and consciousness of stimulus perception, as derived from the discussed empirical measures of human brain waves, needs further confirmation with human ERP, EEG, and brain imaging studies in order to be established as a general guideline for the understanding of brain processes in conscious stimulus perception. The distinction may become also important for the clarification of the use of the terms “awareness” and “consciousness” in the context of consciousness studies in humans (e.g., Morin, 2006). For example, in the following definition of consciousness “*consciousness is the subjective awareness of momentary experience interpreted in the context of personal memory and the present state*” (John, 2003), the first part of the definition (*subjective awareness of momentary experience*) is related to awareness, and the second part, the *interpretation* of *the momentary experience*, is related to consciousness. This distinction between awareness and consciousness will also be useful to guide the search for awareness and consciousness in animals. In the following, we give a strongly condensed review of the main facts that could be helpful for the analysis and interpretation of animal data.

## Awareness and consciousness in animals and their relationship to behavior

### Awareness or, in how far is awareness necessary for stimulus perception and action generation?

It is our own human experience that sensing and responding via reflexes happens without awareness both of the stimuli and the reflex behavior. Examples are the acoustic startle (Koch, 1999) and the vestibulo-ocular reflex (Sadeghi et al., 2010). Such kinds of reflexes can also be conditioned without awareness, for example, when the mammalian subjects are under anesthesia (Ghoneim and Block, 1997). That is, awareness/consciousness are not necessary conditions for sensing, responding, and learning via classical conditioning. Even learning of vocabulary, the formation of declarative memory, can be observed in the non-conscious state of sleep (Ruch and Henke, 2020). Usually, however, explicit (declarative) learning requires awareness/consciousness while implicit (non-declarative) learning does not require awareness/consciousness (e.g., Squire and Knowlton, 1995; Rolls, 2014; Squire and Dede, 2015; Knowlton et al., 2017). Therefore, acquiring knowledge about procedures expressed by skills and habits in humans and animals can and usually do happen without subjects becoming aware that they learn and improve in sensorimotor association and coordination. A further unconscious type of learning concerns priming. A perceived stimulus (independent of the awareness of the stimulus) increases the ability to detect and perceive again this stimulus and/or with this stimulus associated stimuli, also of other modalities, when it/they occur another time (e.g., Squire and Knowlton, 1995; Horga and Maia, 2012; Elgendi et al., 2018). In conclusion, stimulus-guided action generation in the context of implicit learning and priming does not require awareness. Even in a task of declarative visual learning, the formation of working memory and its sustainment over several seconds (up to 4 s in the respective study) can happen unconsciously (Trübutscheck et al., 2017). It is evident that there is broad potential in the kinds of implicit learning, by being primed to respond to certain stimuli, and to work with short-term memory in order to arrange with environmental diversity and variability via adjustment of sensorimotor behavior without awareness.

Perhaps yet more puzzling are reviews presenting evidence that the non-conscious mind can do *everything* in the life of humans and animals (see summaries in Bargh and Morsella, 2008; Hassin, 2013). First of all, this “everything” concerns fixed action patterns that are inherited behavioral routines. For example, walking, flying and swimming in vertebrates are movements coordinated by neural networks in the spinal cord. Awareness or consciousness are not necessary since these movements are possible when the spinal cord is cut from the brain (Barbeau and Rossignol, 1991; Baev and Shimansky, 1992; Martinez et al., 2012). Similarly, fixed action patterns coordinated in the abdominal ganglion of male insects (praying mantis, cockroach) are sufficient for successful copulation without head (Roeder et al., 1960; Matsumoto and Sakai, 2001). Next, this “everything” concerns behavioral routines acquired by implicit (non-declarative) learning or initiated via priming as mentioned above. Finally, this “everything” concerns evolutionary old complex behavior (instincts, goal-directed behavior) based on inherited a-priori knowledge and executed in rather stereotyped ways. Among such behavioral patterns are feeding, drinking, detecting and pursuing prey, navigating, attracting mates, mating, fighting, and care for offspring, all adapted to sustain physiological homeostasis and/or reproductive fitness of a given organism in its ecological niche (examples in e.g., Tinbergen, 1951, 1963; Lorenz, 1981; Wang, 2020). An interesting example of innate, complex prey-catching strategies of a jumping spider has been reviewed by Cross et al. (2020).

Different from reflexes, instincts and goal-directed behavior are *motivated* (e.g., Lorenz, 1981; Epstein, 1982; Wise, 1987; Rolls, 2014), i.e., behavior does not only depend on sensorimotor relationships (Gallistel, 1980, points 1–17 in the summary; Robinson, 1994; Ehret, 1998; Grillner, 2006) but also on internal processes which define the evolved conformity of stimuli and behavior with a biologically relevant goal. These internal processes may be called *motivations*. Therefore, motivation generating neural systems have to be added to and integrated with sensorimotor processing. In a given environment and behavioral context, we observe animals which seemingly select both proper stimuli and fitting actions in order to reach a certain goal according to the actually prevailing motivation. The term motivation stands for the neural output of physiological homeostasis regulation of the body (via food, water, temperature, etc.) able to generate inherited stimulus-controlled actions (e.g., Tinbergen, 1951, 1963; Gallistel, 1980; Lorenz, 1981; Epstein, 1982). How instinctive decision making and action selection in many behavioral contexts such as predator avoidance or navigation can be implemented in evolved brain functions has been shown, for example, by Cisek (2007, 2012), Heinze (2017) and Hoke et al. (2017). Other authors (e.g., Merker, 2007; Denton et al., 2009) stress the contribution of the brainstem of vertebrates for the creation of conscious content and primordial emotion as the origin of consciousness. Likewise, the separation of self-generated sensation from perceived external stimuli via the principle of reafferent processing (von Holst and Mittelstaedt, 1950) shall allow animals to become phenomenally conscious (Merker, 2013; Vallortigara, 2021). Here, we explore neural correlates of the subjective awareness of the content of a given stimulus (phenomenal consciousness according to Block, 2005) which can be understood as the gate to further cognitive handling of the stimulus in the brain. Several examples of the generation of instinctive behavior without implying awareness or even consciousness, in animals can be found in Supplementary Material.

We, as human observers, have to be aware that motivations must not equal *subjectively controlled* urge or intention to behave in a certain way. We know, for example, how little subjective control we demonstrate when we, without awareness, are hustled by subtle advertisement into buying something in the supermarket (Martin and Morich, 2011). Another amazing example is women mate choice, which appears to be governed by unconscious subcortical processes rather than by conscious rational decisions made via implication of the neocortex (Flegr et al., 2019). The extent of unconscious motivated actions in humans is discussed by Lumer (2019). The critical question in our discussion about awareness in animals is whether at all or in how far awareness is a necessary variable in these processes of motivational evaluation and stimulus/action selection in rather stereotyped, stimulus-guided, instinctive or goal-directed behavior. This bottom-up approach in an evolutionary view from subconscious, pre-cognitive behavior (Roth, 1996) to advanced cognition and metacognition is opposite to the usual top-down approach starting with consciousness in humans and then continue with searching for consciousness in behavior of animals, whether or not this is appropriate for the animal species in focus. Similarly, de Waal and Ferrari (2010) proposed a bottom-up approach in the comparison of human and animal cognition. In the same vein, LeDoux (2012) proposed an approach to the understanding of animal emotions. Instead of projecting human emotions such as joy, fear, love, jealousy, etc. onto animals, functions of emotions in animals serving their survival, also in evolutionary terms of fitness, should be reassessed in contexts of human life.

Since the sensorimotor and the central nervous systems need, compared to the rest of the body, high amounts of energy in order to operate adequately, there is evolutionary selective pressure on limiting energy consumption of the brain as far as feasible (de Polavieja, 2002; Niven and Laughlin, 2008; Niven, 2016). For example, for producing high-frequency gamma-wave information exchange between groups of neurons in order to generate awareness and even more consciousness as explained in the first part of this review (compare Figure 2), the energy consumption of the brain would especially be high (Shulman et al., 2009; Chen and Zhang, 2021). By arguments of natural selection, such a high energy consumption of the aware/conscious brain could be an evolutionary meaningful adaptation only if the individuals of a species were not affected by energy shortage in their ecological niche and/or gained a significant advantage in their own or kin reproductive fitness (e.g., West and Gardner, 2013) by having awareness/consciousness as a behaviorally-relevant variable.

### In conclusion

We can state that awareness as subjective experience (for definition, see Introduction) can be absent and may play no role as variable for stimulus selection and generation of behavior as far as stimulus selection and behavior rely only on knowledge acquired via expression of inherited functions, priming, implicit learning, and use of short-term memory. This knowledge concerns stimulus-guided sensorimotor coordination and action selection during the whole course of a behavioral cycle in accordance with the motivational background and with the goals to be reached as defined by the ecological niche of a given animal. When we observe, for example, ants transporting material to their nest, bees visiting flowers for obtaining nectar, fish in agonistic interactions for occupying a territory in a too small aquarium, or female frogs selecting a male according to the acoustic structure of his mating calls, we see in execution adaptive behavior controlled via inherited knowledge. Following the concept of parsimony or Occam’s razor (e.g., Epstein, 1984; Sober, 2015), we can assume that these animals, in the mentioned behavioral contexts, must not be aware of, i.e., must not subjectively experience, the stimuli which their brains perceive and respond to. Awareness, and even more consciousness, can be seen as an energetically expensive luxury that came up in evolution only when it could provide significant advantage in reproductive fitness to the individual and/or its kin.

## Awareness or, do we find neural correlates of awareness in animals?

According to the criteria for reaching the state of awareness derived from brain activity data in humans as mentioned above (ERP peak latencies, selective attention, local gamma-band activity in EEG recordings from higher sensory processing areas), we searched for correlates of awareness in animals. Since animals have brains of various sizes and, possibly, various levels of signal processing, ERP latencies of animals may differ from those of humans, e.g., animals with smaller than human brains may have shorter ERP latencies associated with signaling awareness/consciousness (Siegel et al., 2003; Woodman, 2012). On this background, we selected animal data which allowed a separation of brain waves by our criteria into those that could be addressed as awareness response and those suggesting conscious perception. As a result, we could find correlates of possible awareness in a bird, non-primate mammals and non-human primates. The following examples shall demonstrate this.

### Birds

**FIGURE 3:**
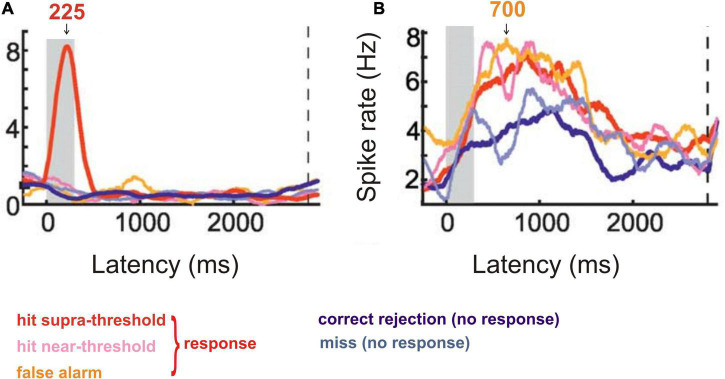
Response behavior of neurons from the crow nidopallium caudolaterale to visual stimuli in a stimulus detection task. **(A)** Cumulative spike rate of an example neuron responding only to a highly visible, supra-threshold stimulus with a response peak about 225 ms after stimulus onset (stimulus duration is shown by the gray area). The neurons of this response type signaled the intensity of the correct stimulus and, thus, the ability to become aware of it. **(B)** Cumulative spike rate of an example neuron responding in the preparation of a response to a perceived (supra-threshold or near-threshold) or allegedly perceived (false alarm) stimulus. The response was trained to be given after a 2800 ms delay from stimulus onset (dashed vertical line in **A** and **B**). Response rates in a latency window of about 380–1000 ms after stimulus onset (see arrow at 700 ms) were significantly higher when the animal responded compared to the cases when it did not respond (correct rejection or miss). The neurons of this type signaled the conscious response preparation. [Modified from Nieder et al. (2020); their Figure 2C (here Figure 3A) and Figure 2E (here Figure 3B)].

Crows were trained to respond to a certain visual stimulus and to ignore a different stimulus in a delayed detection task (Nieder et al., 2020). Recordings from single neurons in the nidopallium caudolaterale, the analogue structure to the mammalian prefrontal cortex (Güntürkün, 2005; Herold et al., 2011; Güntürkün and Bugnyar, 2016), of the behaving crows showed (Nieder et al., 2020) that many neurons discriminated both between absence and presence of the trained visual stimulus (strong response around 225 ms after onset of the trained stimulus; Figure 3A) and between responding and non-responding of the crow in a latency period of about 380–1000 ms (Figure 3B). This neural behavior strongly suggests awareness of the correct stimulus by the early response peak after stimulus onset (Figure 3A), corresponding to a suggested ERP peak with this latency.

### Non-primate mammals, rodents

**FIGURE 4:**
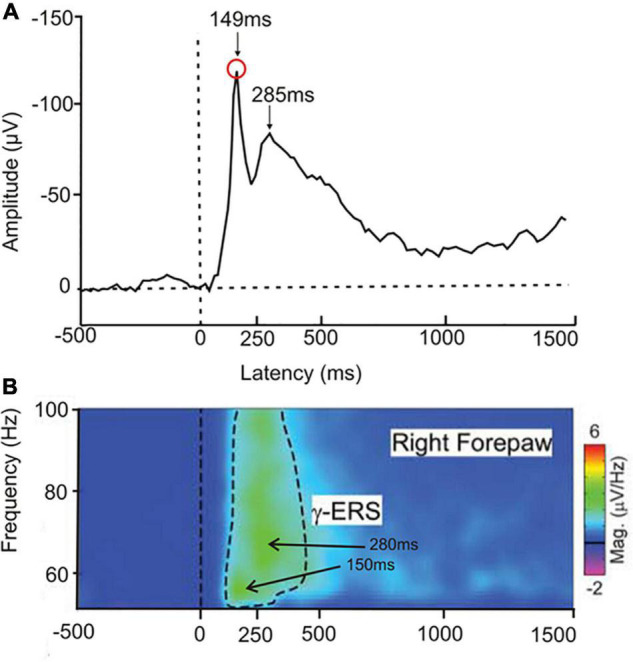
Responses of freely moving rats to a heat stimulus applied to one of the paws. **(A)** Averaged ERP (4 central electrodes on the rat head, 12 animals in the experimental group) in response to a heat stimulus to the right forepaw. Two negativity peaks are visible, one at about 149 ms latency after heat onset, the other at about 285 ms latency. (Modified from Peng et al., 2018; their Supplementary Figure 3). **(B)** EEG group response to a single heat stimulus averaged to display the gamma-band event-related synchronization (γ-ERS) for frequencies of 50–100 Hz. There are two intensity peaks of the gamma-waves, one at about 55 Hz with a 150 ms latency after heat onset, the other at about 65 Hz with a 280 ms latency. The latencies of these peaks in the gamma-band activity correspond closely to the ERP peaks shown in **(A)**. [Modified from Peng et al. (2018); their Figure 2].

In a test on the perception of laser-beam generated local heat spots on front or hind paws (Peng et al., 2018), *rats* showed ERP peaks over the sensory-motor cortex with latencies to heat onset of near 150 and 280 ms (Figure 4A) and high gamma-band EEG cortical waves with intensity peaks at similar latencies (Figure 4B). The peak near 150 ms latency might correspond to awareness, the peak near 280 ms to conscious perception. In rats, similar to human studies (Wyart and Tallon-Baudry, 2008; compare Figure 2), earlier gamma-waves, possibly signaling awareness, had lower frequencies than later gamma-waves, possibly associated with the conscious initiation of a response behavior. The amplitudes of the gamma-waves in the rats correlated positively with the strength of the behavioral response to the heat. The ERP data and the gamma activity together with the behavior suggest awareness of the stimulus and its strength.

Dot motion in the visual field of awake *mice* consistently induced strong gamma-band waves about 200 ms after stimulus onset in the visual cortex both to random (incoherent) motions of the dots and to coherent motions of the dots in one direction. Interestingly, consistent and strong gamma-wave responses both in the visual and frontal cortex were recorded only to the coherent dot motions (Han et al., 2017). This suggests that the mouse could have become aware of the dot motions (visual cortex gamma wave) and, possibly, conscious of a coherent object moving in the visual field (visual and frontal cortex gamma waves).

### Non-primate mammals

**FIGURE 5:**
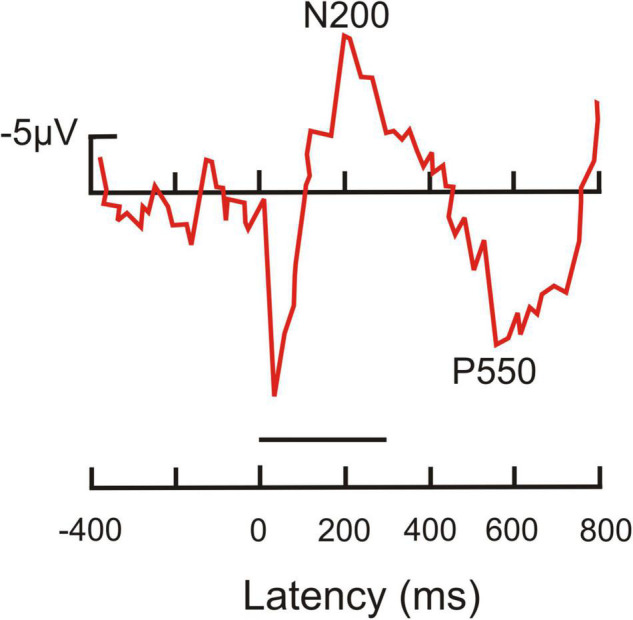
Average ERP recorded from the head of a dolphin in response to a target tone. This tone had a duration of 300 ms (horizontal line above latency axis). It was followed by a second tone signaling conditioned access to a food reward. The ERP shows a prominent negativity peaking near 200 ms latency (N200) after tone onset, and a prominent positivity around 550 ms latency (P550). [Modified from Woods et al. (1986); their Figure 3.2 response to tone #3, target tone].

*Dolphin* ERPs were taken in awake and behaving animals in response to frequent and rare tones which were delivered in a water tank with the animal’s lower jaw being under water to provide adequate listening conditions (Woods et al., 1986). In passive listening and task-related tests, rare tones elicited a negative going ERP peak near 200 ms tone response latency, and a late and broad positive peak around 550 ms latency (Figure 5). The 200 ms peak can be addressed as reflecting awareness, the 550 ms peak conscious perception with response option.

### Non-human primates

Macaque monkeys showed ERP visual awareness negativities with similar properties as humans do, with response latencies at about 170 ms, which is about 50 ms shorter than those of humans in a comparable task (Woodman et al., 2007). In an auditory paradigm, ERPs were present about 200 ms (awareness negativity) and 300 ms (late positivity) after tone onset when monkeys were required to attentively listen to do a task when they heard an odd tone. These potentials were absent when no task was required or a given tone was present during the whole test time (Arthur and Starr, 1984).

Neurons in the prefrontal cortex of awake and behaving macaque monkeys responded in a visual signal detection task with a response peak about 210 ms after stimulus onset when they had perceived a trained stimulus (hit; van Vugt et al., 2018). In another test, monkey prefrontal neurons showed a response peak about 230 ms after stimulus onset to a naturally preferred (not to a neutral) visual stimulus and, in addition, a significant increase in gamma-band activity in the high frequency range above 50 Hz (Panagiotaropoulos et al., 2012). Together with adequate controls, these data indicate awareness of a visually significant stimulus signaled by monkey prefrontal neurons about 200–230 ms after stimulus onset, corresponding to a suggested ERP peak with this latency.

### Cases that need further studies to be settled

#### Fish

Local gamma-band waves have been recorded in the pallium (part of the telencephalon) of the electric fish *Gnathonemus* in response to various sensory stimuli and also as coherent activations in neighboring brain areas (Prechtl et al., 1998). These gamma-waves start with latencies of about 50 ms after stimulus onset. They are accompanied by a large ERP negativity also peaking near 50 ms latency. Although telencephalic gamma-waves, especially corresponding to multimodal processing, may suggest awareness of the stimuli, the response latencies are short and inconclusive. Therefore, further tests, also in combination with behavioral responses, have to show whether possible awareness may have neural correlates in the fish brain.

#### Cephalopods

**FIGURE 6:**
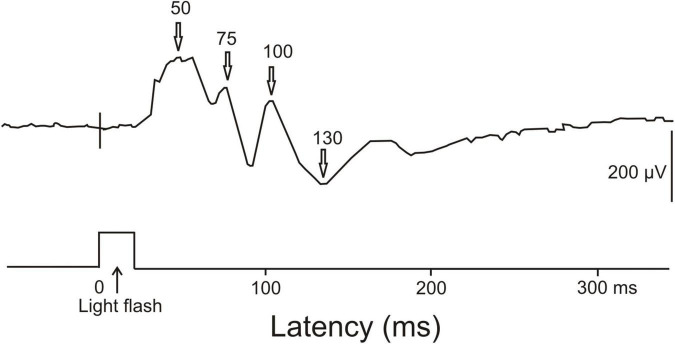
ERP recorded from the central nervous system of a cuttlefish (*Sepia*) in response to a single light flash. The light flash had a duration of 20 ms. The ERP response showed main positivities at about 50, 75, and 100 ms latency and a negativity around 130 ms latency. [Modified from Bullock and Budelmann (1991); their Figure 2, lower part].

Visual ERPs have been obtained from awake unrestrained cuttlefish (Bullock and Budelmann, 1991). The largest ERPs to a 20 ms light flash were found in the anterior part of the median basal lobe or precommissural lobe near the midline. ERPs had positive peaks at about 50 ms, 75 ms and 100 ms, and a broad negative wave around 130 ms after the onset of the light flash (Figure 6). Further late waves could have followed this negativity. On the first view, the early positive waves may correspond to ERPs in the human brain signaling unconscious processing. The late negativity might correspond to the human visual awareness negativity. Since we could not find further ERP studies and, especially, ERP studies combined with behavioral tests for supporting awareness of perception, or EEG studies showing gamma-waves in associative brain centers, we must remain inconclusive about brain activities in cephalopods related to the possible awareness of the animals.

#### Insects

Local field potentials recorded from the mushroom bodies (van Swinderen and Greenspan, 2003) and the central body (Grabowska et al., 2020) of the drosophila brain show high-frequency (20–30 Hz) neural activity when a fly fixates a visual object in the center of its visual field. The amplitude of the 20–30 Hz response is increased when the fly puts selective attention on the visual object, for example, by associating banana smell with the object. The 20–30 Hz activity does not equal but comes close to gamma-band activity in the human brain indicating the possibility of becoming aware. Also, the amplitude increase of the brain activity with an attention increase of the animal, because the object may turn out as a valuable food source, suggests that the fly may have subjectively experienced the object. Further studies have to clarify, however, whether the 20–30 Hz activity in the mushroom bodies or the central body and the activity increase by smell association is due to automatic sorting for positive or negative action generation guided by prepared learning of certain stimuli conditional by evolution (Webb, 2012; Smid and Vet, 2016) or reflects action planning due to free associative learning. Further, in the case of associative learning, the use of long-term memory for the association (vision-smell) has to be tested in order to make sure that the attention on the visual object is not based on inherited bottom-up stimulus-specific attentional mechanisms possibly supporting prepared learning (Dunlap and Stephens, 2014; Smid and Vet, 2016).

### In conclusion

The example data of brain activity from birds and mammals provide evidence to suggest that, besides humans, animals of these groups can become aware of, i.e., can subjectively experience, sensory stimuli. The brain activity data from fish, cephalopods, and insects (drosophila), provide some hints for possible awareness. They are, however, inconclusive so far, because further studies have to clarify in how far the brain activities represent inherited knowledge and/or the results of priming, implicit learning and short-term memory, as explained in the previous section of this review.

## Consciousness or, do we find neural correlates of consciousness in animals?

Some previously mentioned ERP studies in the context of signaling awareness (late negativities) also showed late positive ERP peaks (compare Figures 3–5) suggesting conscious stimulus perception with the option of responding. These late ERP peaks concern a *bird*, the crow visual study with late spike-rate maxima corresponding to suggested ERP peaks with this latency (Nieder et al., 2020), *rats* (ERPs and gamma-waves to heat stimuli, Peng et al., 2018), *dolphins* (auditory ERPs, Woods et al., 1986), and *macaque monkeys* (auditory ERPs, Arthur and Starr, 1984).

Paller (1994) reviewed ERP studies with further mammals (cats, dogs, rabbits, squirrel monkeys), specifically concerning late positive ERP responses such as P300 to a variety of stimuli in passive and task-related perception tests. He discussed the animal data in relation to those available from humans and found a large variability in P300-like ERP waves in animals. This variability concerned peak latency, peak polarity, relative peak amplitude, the relationships of P300-like peaks to other ERP waves, the P300-like dependence on task requirements, and on the control of the attention of the animals in the tests. To ensure optimal comparability of animal and human data, the data sets should be taken with the same kind of stimulus paradigm and with the same or similar (adapted to the animal’s behavioral abilities and preferences) task requirements (Paller, 1994). This may be difficult experimentally, however, not impossible as the reported results in our review demonstrate.

### In conclusion

Similar to the conclusions of the awareness part, the example data of brain activity from a bird and mammals provide evidence supporting the hypothesis that, besides humans, animals of these groups may become conscious of the perceived sensory stimuli. Further studies are, however, necessary, especially combining behavioral responses with neural activity recordings, to clarify consciousness not only in mammals and certain bird species but also, and especially, in the other animal groups mentioned.

## General discussion

### Besides humans, mammals and birds may perceive stimuli with awareness and consciousness

By making a distinction between awareness and consciousness in defining these terms (see Introduction), we could separate NCCs of these attributes of brain function in data of brain activities related to stimulus perception and responding in humans. Valuable NCCs were found in recordings of ERPs and EEG gamma-waves from which correlations mainly between latencies of EEG peaks, latencies of strong gamma-wave occurrence locally or globally, and behavioral indicators of awareness and consciousness could be extracted. Remarkably, these correlations were not only consistent among numerous studies from humans but could also successfully be applied to data sets from animals. Thus, our comparative analysis identified measures of brain activity that can be used as neural markers indicating potential awareness and consciousness while perceiving sensory stimuli, at least in mammals and birds.

To be careful, for this review we could locate only few appropriate data samples for mammals, and only a single one for birds. That is, our conclusions are promising, however, tentative, and need more experimental support with data from many more bird and mammalian species in order to be generalized. With further scientific validation, these common neural markers can be useful in well controlled and objectifiable animal tests in the laboratory to get a better understanding of how awareness of and consciousness about perceived stimuli may be generated by and represented in mammalian and bird brain activity.

We saw that awareness and consciousness in stimulus perception of humans depended on the concomitance of selective attention with the stimuli. Selective attention may even be necessary for awareness to be realized (Dehaene and Naccache, 2001; Cohen et al., 2012). Selective attention has been described for mammals (e.g., Rizzolatti, 1983; Thiele and Bellgrove, 2018), non-mammalian vertebrates (e.g., Krauzlis et al., 2018), cephalopods (e.g., Schnell et al., 2021) and arthropods (e.g., de Bivort and van Swinderen, 2016; Nityananda, 2016; Bruce et al., 2021). This presence of selective attention in various animal groups supports the notion that members of these groups may become aware or even conscious of perceived stimuli.

Together with several other brain functions, selective attention has been observed with an advantage of expression in the left brain-hemisphere of vertebrates (e.g., Ehret, 2006; Rogers and Vallortigara, 2015; Güntürkün et al., 2020). Because of the crossed projections of sense organs to their higher processing areas in vertebrate brains, this suggests that sensory stimuli (auditory, visual, somatosensory) picked up from the right side of the body and processed preferentially in the left brain-hemisphere may reach awareness at lower perception thresholds or more intensively than those perceived via the left body side. This is a broad field open for further research.

The ERP data from humans showed awareness negativities lateralized in the brain, i.e., their amplitudes were highest contralateral to the side of the sensory input. We did not see this phenomenon reported in the animal studies. In future ERP studies on animals, it would be highly informative to analyze ERPs separately for the left and right brain hemisphere. Lateralized ERP peaks with the appropriate latencies could be related to awareness while later peaks related to consciousness are expected to have the same amplitudes on each hemisphere because of the more global brain activation associated with conscious perception. This is another promising field of research.

Our analysis did not concern consciousness in contexts of cognition-based action generation and higher-level cognitive functions such as introspective awareness and metacognition, as indicated in the Introduction. If we considered behavioral evidence for animal consciousness in the context of higher-level cognitive functions, we could have predicted the possibility of awareness/consciousness in several species of mammals and birds as suggested by (a) self-recognition in great apes (Gallup and Anderson, 2020), and possibly in elephants, dolphins, magpies (Derégnaucourt and Bovet, 2016; de Waal, 2019), (b) tool manufacturing in apes, monkeys (macaque, cebus), mammals such as beaver, elephant, dolphin, and several bird species such as crows and parrots (Bentley-Condit and Smith, 2010), (c) other examples of metacognition or insight in problem-solving tasks as found in apes (Jolly, 1991; Emery and Clayton, 2004), macaques (Hampton, 2001; Chang et al., 2017), marmoset monkeys (Burkart and van Schaik, 2020), rats (Foote and Crystal, 2007), corvids and parrots (Heinrich, 2000; Emery and Clayton, 2004; Baciadonna et al., 2021). These behavioral data derived from tests of higher cognitive functions in animals are in general agreement with the neural data derived from ERP and gamma-wave brain activity what possible awareness/consciousness in animal groups are concerned. According to these data, species of mammals (including humans) and certain bird species show the ability to become aware and conscious both in stimulus perception tasks and in tasks requiring *thinking over* before starting to do something.

### Can we assess the data from fish, cephalopods and insects?

The criteria which we used to evaluate ERP and gamma-wave data did not lead to conclusive suggestions about awareness and consciousness in stimulus perception by heterothermic vertebrates and invertebrates. Responsible for this inconclusiveness is the obvious paucity of adequate neurophysiological data recorded in behaviorally relevant contexts. Therefore, we may consider in addition behavioral evidence for animal consciousness in the context of higher-level cognitive functions according to the above-mentioned aspects (self-recognition, tool manufacturing, metacognition or insight in problem solving tasks).

Here, we could find possible self-recognition in a fish species, the cleaner wrasse, via mirror tests (Kohda et al., 2019, 2022; see however, reservations about the significance of mirror tests for assessing self-recognition by de Waal, 2019), but no tool manufacturing and no metacognition or insight in problem solving tasks. In comprehensive reviews about cognition in cephalopods (Schnell et al., 2021) and insects, especially bees (Giurfa, 2015), metacognition-*like* use of associative memory has been suggested. In addition, episodic-*like* memory has been reported in studies of learning to manage food access in cuttlefish (Jozet-Alves et al., 2013) and bees (Pahl et al., 2007). Since formation and recall of episodic memory usually requires awareness/consciousness in humans (Squire and Knowlton, 1995; Squire and Dede, 2015; Knowlton et al., 2017), data on episodic memory in cephalopods and insects would support the suggestion that species in these groups of animals may at least become aware of the stimuli they perceive, i.e., which stimuli, where, and when. The ability for and the realization of associative learning in contexts of stimulus perception and action generation is, however, not per se sufficient for assuming that awareness/consciousness may be important factors in the learning processes (e.g., Dehaene and Naccache, 2001; Webb, 2012). Therefore, further recordings of brain activity in behaving animals may provide decisive evidence for the presence of awareness/consciousness in a cognitive task, for example when changes of brain activity correlate with the gating of functional connectivity between centers of associative signal processing while the animal directs selective attention to certain signals in order to determine their significance (arising from information in long-term memory) in a given task.

### What can we infer from the data with regard to the evolution of awareness and consciousness?

Current evidence suggests that, in addition to instinctive routines, arthropods, cephalopods and vertebrates have access to brain processes of selective attention that can be directed toward stimuli, so that the stimuli can, in principle, be subjectively experienced. Animals may become aware of the stimuli with the option to consciously respond. Metzinger (2009; cited in Le Neindre et al., 2017) expressed the relationship between attention and consciousness as …*in essence, consciousness is the space of attentional agency*. Transformed to the substrate of the brain, we can reformulate: Consciousness is the spatial and temporal space of brain activity that can actually be activated by selective attention. Awareness can be integrated in this metaphor as *a spot* in consciousness, i.e., as a spot in the space of attentional agency or, transformed, as a spatial and temporal spot in the space of potential brain activity to be activated by selective attention.

**FIGURE 7:**
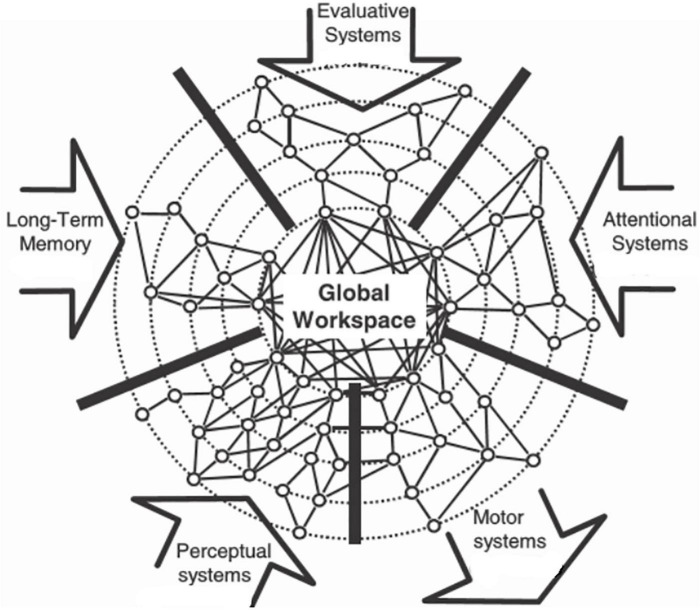
Global Neuronal Workspace (GNW) hypothesis for the generation of consciousness in the brain. The input arrows to the global workspace show systems (perceptual, long-term memory, evaluative, attentional) which can contribute to an interactive processing with the result of the generation of awareness of the content of stimuli and consciousness about the preparation and execution of behavior (motor systems output arrow). [Modified from Dehaene and Changeux (2011); their Figure 6, upper right panel].

These metaphors for awareness and consciousness can be used for a straightforward understanding of some aspects concerning the evolution of awareness and consciousness in animals (awareness and consciousness as defined in the Introduction). Irrespective of the actual anatomical organization of the central nervous system of a given animal, the functional organization should be compatible with the Global Neuronal Workspace hypothesis (GNW; Dehaene and Changeux, 2011; Figure 7) describing the interactional processes in large neuronal networks in order to produce consciousness. According to our definition of awareness and consciousness, the interaction of perceptual, long-term memory and attentional systems could produce awareness of stimulus content. The interaction of evaluative systems, long-term memory, and attentional systems could produce consciousness for the preparation of actions. Accordingly, we predict that animals without systems of attention, especially of selective attention, and without long-term memory will not experience awareness of the content of something or consciousness about something to do.

With the above metaphoric transformation of Metzinger’s (2009) relationship between consciousness and attention to the brain and to the GNW, we can propose that animals with an inherited possibility to couple very different stimuli of one or several modalities, i.e., a broad input of perceptual systems to be coupled with memory in the GNW, to a single type of response behavior – this is the spot in Metzinger’s space of attentional agency – may *become aware of the stimuli*. Animals with an inherited possibility to couple one stimulus or stimulus context with several types of response behavior, i.e., a broad network of adjustment of motor systems to the processing in evaluative systems and long-term memory – this is the wide space of attentional agency – may *become conscious of the stimulus*. An example of the first case: Awareness of stimuli relates to a spectrum of food items, the abundance and distribution of which may vary across time (seasons) and/or structure of the environment, but all have been learned to serve as potential food and could initiate behavior for getting access to the food and finally eating it. An example of the second case: Consciousness of response options to a given stimulus relates to a conspecific in a social group with which the subject can have several types of interactions (friendly ones, agonistic ones, etc.) of learned outcome. Therefore, in animals with rich sensory input capacities (various and interacting sensory systems) and rich and variable capacities of behavioral output, awareness may have evolved to serve stimulus detection, identification, integration and attribution of stimuli in order to generate adaptive response behavior, and consciousness may have evolved to serve the selection of an adaptive response or an adaptive action in a certain context given that there are several response/action options available. *Serving* in the case of awareness means opening a time window during unaware stimulus processing in order to allow adequate coupling of stimulus information with information about the animal’s state for the preparation of storing this combined information in long-term memory to be used later in comparable situations. *Serving* in the case of consciousness means opening a time window for the evaluation of conditions (actual motivational tendencies, actual environmental conditions, content of long-term memory related to the actual experience) in order to allow selection, adjustment and control of the response/action for optimal adaptivity with regard to genetic fitness. Without addressing awareness and consciousness separately, Keller (2014) in discussing the possible evolutionary function of consciousness in information processing in the olfactory system, proposed a very similar conclusion for consciousness as we did (see above), namely …*the evolutionary function of conscious information processing [is] to guide behaviors in which the organism has to choose between many possible responses*.

The analysis of ERP and EEG data of the human brain has clearly shown the time windows associated with awareness or consciousness (see Figures 1, 2). After stimulus onset, first ERP peaks up to about 200 ms latencies indicated non-conscious signal processing followed by the ERP awareness peak and the onset of gamma-band activity associated with the generation of awareness. Even later ERP peaks and onset of global gamma-band activity indicated conscious preparation of behavior. The actual latencies of ERP peaks and gamma-wave occurrence in animals may differ from those in humans according to the sizes of the animals’ brains and the number of evaluative levels to be passed in a GNW analogue. However, we suggest that this stimulus-activated temporal sequence of brain activity from non-conscious to aware to conscious in adult humans reflects the possible evolution in various animal phyla from non-conscious signal processing and acting to becoming aware of signals and, finally, toward consciousness of acting.

Since awareness and consciousness reflect *optional* brain activity added with high energy costs to the basic, non-conscious signal processing, further animal studies should consider this energy aspect as an important factor of natural selection in the evolution toward possible context-dependent awareness/consciousness of a given species. A recent review (Chen and Zhang, 2021) framed this new field of research.

## Conclusion

Neural markers in human ERPs and gamma-wave brain activity can differentiate between awareness of stimulus perception and consciousness in response generation. These markers can successfully be used to assess the possibility of becoming aware of a stimulus and of responding consciously in species of mammals and birds. These conclusions are promising, however, tentative, and need more experimental support with data from many more bird and mammalian species in order to be generalized. The results of applying these markers to data from fish, cephalopods and insects remained inconclusive. Since the original data have not been recorded with the aim of testing awareness/consciousness in the animals, the experimental designs were not optimized for giving answers to these questions of our interest and, thus, may have led to inconclusive results. Besides methodological reasons for preventing conclusive statements about awareness/consciousness, for example the experimental control of selective (focused) attention which seems necessary for awareness and consciousness to be generated, biological conditions have to be studied in an evolutionary perspective in order to predict in how far subjective experiences would be helpful and advantageous to increase the reproductive fitness of members of a given species. In other words, studies should investigate the ability to display selective attention and long-term memory in a given species, and study whether awareness and/or consciousness are necessary vital factors in the natural lifecycle of that species. The aware and conscious brain causes high energy costs which, in an evolutionary approach, can be tolerated only if a significant gain in reproductive fitness for the individual and/or its kin can be achieved by becoming aware or conscious, at least in some behavioral contexts, with promising fitness rewards.

## Data availability statement

The original contributions presented in this study are included in the article/Supplementary Material, further inquiries can be directed to the corresponding author/s.

## Author contributions

GE initiated the review, screened and analyzed the material, and wrote the manuscript. RR initiated the project, contributed ideas, and read and approved the text. Both authors contributed to the article and approved the submitted version.

## Conflict of interest

The authors declare that the research was conducted in the absence of any commercial or financial relationships that could be construed as a potential conflict of interest.

## Publisher’s note

All claims expressed in this article are solely those of the authors and do not necessarily represent those of their affiliated organizations, or those of the publisher, the editors and the reviewers. Any product that may be evaluated in this article, or claim that may be made by its manufacturer, is not guaranteed or endorsed by the publisher.
