# Supplementary material for: Awareness and consciousness in humans and animals – neural and behavioral correlates in an evolutionary perspective
Source: Front Syst Neurosci. 2022 Jul 14;16:941534. doi: 10.3389/fnsys.2022.941534 (PMC9331465; doi:10.3389/fnsys.2022.941534)
Supplement: Supplementary file 1 [file Data_Sheet_1.docx]

**Supplement 1: Examples of behavioral regulation without awareness as necessary variables**

There are abundant examples of changes in sensorimotor behavior of animals in many taxonomic groups that we, as human observers, may assume to be specifically motivated or even actively intended by the aware animal. Often, however, inherited behavioral changes of or between fixed action patterns happen, caused by changes of the stimulus context and/or by physiological changes in the homeostasis regulation of the body. Six examples shall illuminate instinctive or goal directed behavioral adjustments that do not require awareness.

Cnidaria are without brains. Various neural networks have been shown to be activated by different stimuli to coordinate the behaviors feeding or swimming in **jellyfish** (Pallasdies et al., 2019; Weissbourd et al., 2021). Switching between behaviors has been observed by antagonistic coupling between networks as shown in the polyp *Hydra* (Dupre and Yuste, 2017). Since swimming and feeding are fixed action patterns, which seem to antagonistically exclude each other (explicit memory absent), absence of awareness can generally be assumed in cnidaria (polyp, jellyfish).

**Nematode worms** have a nervous system mainly consisting of a head ganglion and a ventral and dorsal cord with 302 neurons altogether in the most studied species, *Caenorhabditis elegans* (White et al., 1986). Behavioral switching between roaming (fast moving) and dwelling (stationary) states in this worm is generated by reciprocal inhibitory coupling of two neurons, each responsible for roaming or dwelling, respectively (Ji et al., 2021). Since roaming and dwelling states, which can be activated spontaneously, are fixed action patterns with the switch explained by simple reciprocal inhibition between two neurons (explicit memory absent), awareness must not be assumed. In the same animal, the learned switch from feeding on certain bacteria to avoiding the intake of the bacteria has been studied. The learned avoidance is expressed by a chemical change in a group of sensory cells and this change, causing the avoidance behavior, is epigenetically passed on to next generations (Moore et al., 2019). Thus, food selection depends on chemical sensitivity of sensory cells at the head and, if this sensitivity is changed via learning and kept as memory, the signals to the brain are different and therefore automatically lead to different behavior (food rejection instead of food acceptance). Behavioral changes are initiated by the sensors of the stimuli, in other words, the stimuli determine what we, as observers, would call a motivated behavior. Since there is no subjectively controlled change in motivation, only a stimulus-controlled behavioral change, awareness must not be assumed.

The **electric fish**, *Eigenmannia*, responds to the electric organ discharge of conspecifics with either increasing or decreasing its own discharge frequency depending on whether the other’s frequency is a bit lower or higher, respectively (Heiligenberg, 1991). This inherited change of a fixed action pattern is called jamming avoidance response, comparable with an orienting response. It is coordinated in the brainstem and does occur automatically without learning.

**Toads** visually discriminate potential prey from potential enemies and behave accordingly (turning towards or away from the object). The discrimination is based on inherited knowledge regarding shape, size and movement-in-space parameters of prey versus enemy, and regarding the stimulus-induced motivational changes which are coordinated by antagonistically coupled neural networks in the midbrain, pretectum, and thalamus (Ewert, 1987). A naive toad, ready for responding to visual stimuli from the environment, does prey/enemy discrimination routinely without learning, so that subjective awareness of the stimuli must not be assumed.

**House mouse** mothers have inherited knowledge or are primed immediately after birth of their pups to respond to ultrasounds of lost pups with maternal search and pup-retrieval behavior. The access to maternal *motivation* in the mothers is generated mainly via hormonal changes during pregnancy and giving birth (e.g., Rosenblatt and Siegel, 1981). The classification of ultrasounds (categorical perception) in relevant and irrelevant ones for the release of maternal behavior (Ehret and Haack, 1982, Ehret, 1992) is based on sound processing in the auditory midbrain inferior colliculus (Ehret and Merzenich, 1985, 1988; Egorova and Ehret, 2008; Egorova et al., 2020) and does not require learning (Ehret, 1987). Similarly without learning, house mouse mothers, immediately after delivery and in a nursing context in the nest, rank low-frequency sounds (wriggling calls) of their pups in rather relevant or irrelevant ones (non-categorical perception) for the release of three different maternal responses (Ehret and Riecke, 2002; Geissler and Ehret, 2002). Stimulus evaluation is again based on sound processing in the auditory midbrain (Akimov et al., 2017; Geissler et al., 2018). These cases reflect inherited/primed stimulus discrimination and inherited stimulus-guided maternal motivational change in order to release adequate responses in a given maternal context. Subjective awareness of stimulus properties and of the own physiological state (being maternal) is not necessary for responding in a biologically meaningful way.

**Honey bees** outside their hive are motivated to search for food (nectar). They can use visual landmarks as indicators for getting to a food source in the near neighborhood. They can learn to discriminate on their way to the food source between two guiding visual landmarks, an irrelevant one and the other referring to the food source because it is also the mark at the source (Zhang et al., 2005). This learning of explicitly discriminating two visual patterns uses short-term memory (within about 6 s) and, therefore, may not require awareness.

**References**

Akimov, A.G., Egorova, M.A., and Ehret, G. (2017). Spectral summation and facilitation in on- and off-responses for optimized representation of communication calls in mouse inferior colliculus. *Eur. J. Neurosci.* 45, 440-459. doi: 10.1111/ejn.13488

Dupre, C., and Yuste, R. (2017). Non-overlapping neural networks in *Hyla vulgaris*. *Curr. Biol.* 27, 1085-1097. doi: 10.1016/j.cub.2017.02.049

Egorova, M., and Ehret, G. (2008). Tonotopy and inhibition in the midbrain inferior colliculus shape spectral resolution of sounds in critical bands. *Eur. J. Neurosci.* 28, 675-692.

Egorova, M.A., Akimov, A.G., Khorunzhii, G.D., and Ehret, G. (2020). Frequency response areas of neurons in the mouse inferior colliculus. III. Time-domain responses: constancy, dynamics, and precision in relation to spectral resolution and perception in the time domain. *PLoS ONE* 15(10):e0240853. doi: org/10.1371/journal.pone.0240853

Ehret, G. (1987). Left hemisphere advantage in the mouse brain for ultrasound recognition in a communicative context. *Nature* 325, 249-251.

Ehret, G. (1992). Categorical perception of mouse-pup ultrasounds in the temporal domain. *Anim. Behav.* 43, 409-416.

Ehret, G., and Haack, B. (1982). Ultrasound recognition in house mice: Key-stimulus configuration and recognition mechanisms. *J. Comp. Physiol. A* 148, 245-251.

Ehret, G., and Merzenich, M.M. (1985). Auditory midbrain responses parallel spectral integration phenomena. *Science* 227, 1245-1247.

Ehret, G., and Merzenich, M.M. (1988). Complex sound analysis (frequency resolution, filtering and spectral integration) by single units in the inferior colliculus of the cat. *Brain Res. Revs.* 13, 139-163.

Ehret, G., and Riecke, S. (2002). Mice and humans perceive multiharmonic communication sounds in the same way. *Proc. Natl. Acad. Sci. USA* 99, 479-482. doi: 10.1073/pnas.012391999

Ewert, J.P. (1987). Neuroethology of releasing mechanisms: Prey catching in toads. *Behav. Brain Sci.* 10, 337-405.

Geissler, D.B., and Ehret, G. (2002). Time-critical integration of formants for perception of communication calls in mice. *Proc. Natl. Acad. Sci USA* 99, 9021-9025. doi: 10.1073/pnas.122606499

Geissler, D.B., Weiler, E., and Ehret, G. (2018). Adaptation and spectral enhancement at auditory perceptual boundaries – Measurements via temporal precision of auditory brainstem responses. *PLoS ONE* 13(12): e0208935. doi: org/10.1371/journal.pone.0208935

Heiligenberg, W.F. (1991). *Neural Nets in Electric Fish*. Cambridge Mass: MIT Press.

Ji, N., Madan, G.K., Fabre, G.I., Dayan, A., Baker, C.M., Kramer, T.S., et al. (2021). A neural circuit for flexible control of persistent behavioral states. *eLife* 10:e62889. doi: 10.7554/eLife.62889

Moore, R., Kaletsky, R., and Murphy, C.T. (2019). Piwi/PRG-1 argonaute and TGF-ß mediate transgenerational learned pathogenic avoidance. *Cell* 177, 1827-1841. doi: 10.1016/j.cell.2019.05.024

Pallasdies, F., Goedeke, S., Braun, W., Memmesheimer, R.-M. (2019). From single neurons to behavior in the jellyfish *Aurelia aurita*. *eLife* 8:e50084. doi: 10.7554/eLife.50084

Rosenblatt, J.S., and Siegel, H.I. (1981). “Factors governing the onset and maintenance of maternal behavior among nonprimate mammals. The role of hormonal and nonhormonal factors”, in *Parental Care in Mammals*, eds D.J. Gubernick, and P.H. Klopfer (New York: Plenum Press), 13-76.

Weissbourd, B., Momose, T., Nair, A., Kennedy, A., Hunt, B., and Anderson, D.J. (2021). Functional modules within a distributed neural network control feeding in a model medusa. *Cell* 184: 5854-5868. doi: 10.1016/j.cell.2021.10.021

White, J.G., Southgate, E., Thomson, J.N., and Brenner, S. (1986). The structure of the nervous system of the nematode *Caenorhabditis elegans*. *Phil. Trans. R. Soc. Lond. B* 314, 1-340.

Zhang, S., Bock, F., Si, A., Tautz, J., Srinivasan, M.V. (2005). Visual working memory in decision making by honey bees. *Proc. Natl. Acad. Sci USA* 102, 5250-5255. doi: 10.1073/pnas.0501440102
